# Emotional Eating among Ghanaian University Students: Associations with Physical and Mental Health Measures

**DOI:** 10.3390/nu15061526

**Published:** 2023-03-22

**Authors:** Mary Amoako, Felicity Amoah-Agyei, Chen Du, Jenifer I. Fenton, Robin M. Tucker

**Affiliations:** 1 Department of Biochemistry and Biotechnology, Kwame Nkrumah University of Science and Technology, Kumasi 00233, Ghana; 2 Department of Food Science and Human Nutrition, Michigan State University, 469 Wilson Rd., East Lansing, MI 48824, USA

**Keywords:** eating behaviors, TFEQ-18, emotional eating, university students

## Abstract

Eating behaviors are a set of cognitive processes that influence dietary decision making and, thus, overall health. Some of the most studied eating behaviors are those characterized by the Three Factor Eating Questionnaire-18 (TFEQ). The TFEQ examines three eating behaviors: emotional eating (EE), uncontrolled eating (UE), and restrained eating (RE). While frequently used, there is little information characterizing these eating behaviors in the Ghanaian population. This cross-sectional study describes EE, UE, and RE behaviors in a university student population (n = 129) in Ghana. Of the three behaviors, EE was the only one associated with any of the health outcomes in this study: BMI for males (r = 0.388, *p* = 0.002) and anxiety (r = 0.471, *p* < 0.001, higher score is less desirable), and sleep quality (r = 0.464, *p* < 0.001, higher score is less desirable) for females. Overweight and obese females reported significantly higher EE scores compared to healthy weight females (35.7 ± 23.7 vs. 11.9 ± 15.6, *p* = 0.002). No such observation was observed among overweight and obese males (*p* > 0.05). EE, UE, and RE scores did not differ between males and females. While this study provides important information about the eating behaviors of Ghanaian university students and allows for comparison to students from other cultures, future work must develop culturally relevant tools for the Ghanaian population.

## 1. Introduction

Eating behaviors are a set of cognitive processes that influence dietary decision making [1]. Because dietary decisions predict the risk of developing diet-related chronic diseases, understanding eating behaviors improves our ability to prevent and treat these conditions. The effects of dietary intake on physical health have been extensively studied, but more recent research suggests that dietary choices also affect or are influenced by mental health [2,3]. Thus, exploring eating behaviors can provide insight into a wide range of potential health outcomes.

Some of the most studied eating behaviors are those represented by the Three Factor Eating Questionnaire (TFEQ) [4,5,6]. The latest version of the TFEQ, the TFEQ-18, is an 18-question survey that examines three eating behaviors: emotional eating (EE), uncontrolled eating (UE), and restrained eating (RE) [4]. EE occurs when an individual eats in response to emotional cues. Typically, the foods selected when engaging in EE are calorically dense [7]. UE refers to a loss of control when eating, leading to over consumption. RE is the act of limiting intake based on considerations of body weight and/or weight gain, rather than based on hunger and fullness cues. Rather than a global score, the three subscale scores for EE, UE, and RE are typically reported.

The eating behaviors represented by the TFEQ have been associated with undesirable health outcomes and behaviors in many studies across diverse populations, including university students in countries like China, Thailand, and Malyasia [8,9,10]. Outcomes associated with TFEQ behaviors include, but are not limited to: BMI, hypertension, and cardiovascular disease [11,12,13,14,15,16], as well as food choices [4]. While the number of studies using the TFEQ-18 to examine eating behaviors is high, few studies have explored its utility in African countries and, to our knowledge, only one study focused on Ghanaian individuals [17].

This study sought to characterize eating behaviors using the TFEQ-18 in a university student population in Ghana. Relationships between the three TFEQ-18 subscales, EE, UE, and RE, and physical health outcomes were evaluated. Further, because these eating behaviors are cognitive processes, the relationships between TFEQ-18 subscales and other cognitive operations, like stress perception, resilience, and anxiety were investigated. Because TFEQ responses may vary by BMI status [18], exploratory analyses to determine differences in outcomes based on BMI status were also undertaken.

## 2. Materials and Methods

### 2.1. Study Design

Data were collected between October 2020 and January 2021 using the online survey platform Qualtrics (Provo, UT, USA). Students attending the Kwame Nkrumah University of Science and Technology (KNUST), Kumasi, Ghana were invited to participate. At this time, students were studying at home due to the COVID-19 pandemic. The study was approved by the Committee of Human Research Publication and Ethics (CHRPE), School of Medical Sciences, KNUST and informed consent was obtained from each participant prior to initiating the study. The tools listed below were used to collect data.

### 2.2. Demographics and Body Mass Index (BMI)

Respondents self-reported demographic information (age, class standing, enrollment status, i.e., full vs. part-time) as well as height and weight. BMI (kg/m^2^) was calculated.

### 2.3. Eating Behaviors

As discussed above, the TFEQ-18 was used to assess EE, UE, and RE [4]. Subscale scores were transformed into continuous scores using the formula [(raw score − lowest possible raw score)/possible raw score range) × 100]. As a result, possible scores range from 0–100. Higher subscale scores indicate a greater risk for engaging in the behavior of interest.

### 2.4. Dietary Patterns

The Starting the Conversation food frequency questionnaire (STC) [19] was used to assess dietary habits. Questions cover a variety of topics including fruit and vegetable as well as snack food consumption. Scores range from 0–16, with higher scores indicating more undesirable dietary habits.

### 2.5. Perceived Stress

Perceived stress was assessed using the Perceived Stress Scale-10 (PSS-10) [20]. The PSS-10 surveys stress over the past month. Scores range from 0–40.

### 2.6. Financial Stress

The University Student Financial Stress Assessment (USFSA) tool examined financial stress [21]. While developed for the US student population, the questions are, in general, applicable to students studying in Ghana. Questions include: “I feel stressed about my personal finances in general; I worry about being able to pay monthly expenses; and I worry about having enough money to pay for school”. Responses range from 1–4 (strongly disagree to strongly agree). Additional questions include: “How much stress does the total amount of money you owe cause you; how much stress does credit card debt cause you; and how much stress does student loan debt cause you?” Responses for these questions include numerical values 1–6 which reflect answers of “does not apply/no debt” to “extreme amount”. Possible scores range from 6–30; higher scores are indicative of greater stress.

### 2.7. Resilience

The Brief Resilience Scale (BRS) was used to assess psychological resilience [22]. Possible scores range from 0–5. A higher score is consistent with greater resilience.

### 2.8. Anxiety

Anxiety levels were determined using the Generalized Anxiety Disorder Screener (GAD-7) [23]. The GAD-7 assesses anxiety symptoms over the past two weeks. Scores range from 0–21.

### 2.9. Sleep

Sleep duration and quality were assessed using the Pittsburgh Sleep Quality Index (PSQI). The PSQI measures sleep quality and habitual sleep duration over the past month [24] and has been used to evaluate sleep measures in many populations, including university students in Ghana [25]. Possible PSQI scores range from 0–21. Higher scores suggest worsening sleep quality; whereas, a score of ≥5 indicates poor sleep quality [24]. Participants were also asked how many hours of sleep they usually achieved during the weekdays as well as on the weekends. Average sleep duration was calculated based on the following calculation: ((weekday sleep duration × 5) + (weekend sleep duration × 2))/7.

### 2.10. Data Analysis

Data were analyzed using SPSS v. 28.0.1.1 (IBM Corp, Armonk, New York, NY, USA). Independent t-tests were applied to identify both differences by gender, since previous work suggests males and females experience EE, UE, and RE differently [26,27,28,29] and differences by BMI classification (BMI ≥ 25.0 kg/m^2^ considered overweight/obese). Pearson product moment correlations were used to identify the strength and directions of relationships between TFEQ subscale scores and the outcome or behavior of interest. A median split was used to divide participants into two groups, “low” and “high” EE, UE, or RE. Fischer’s exact test was employed to check for differences between groups. Cronbach’s alpha was calculated to assess reliability of the three subscales [30]. Given the number of comparisons, a False Discovery Rate (FDR) of 0.05 was applied [31].

## 3. Results

A total of 129 students (male = 72, female = 57) completed the study. The average age was 21.3 ± 2.4 y, and the majority (88.3%) of students were undergraduates. Only 4 respondents (3.1%) indicated they were enrolled in university on a part-time basis. Cronbach’s alpha for EE, UE, and CR were 0.853, 0.601, and 0.371, respectively.

After FDR correction, there were no differences between males and females for the outcomes of interest in this study (Table 1).

Although mean scores did not differ by sex, associations between EE and variables of interest did. Among males, correlations were significant between EE and UE (r = 0.503, *p* < 0.001) as well as EE and BMI (r = 0.388, *p* = 0.002). Among females, significant associations were also observed between EE and UE (r = 0.567, *p* < 0.001), EE and anxiety (r = 0.471, *p* < 0.001, higher score is less desirable), and EE and sleep quality (r = 0.464, *p* < 0.001, higher score is less desirable).

Associations between UE and RE differed from those observed with EE. Among both males and females, UE was associated with EE, as discussed above. No other associations survived FDR analysis. Associations between RE and variables of interest were not observed for either sex.

The only outcome that varied by BMI category was EE in females. Overweight and obese females (n = 14, 24.6% of the BMIs sampled) reported significantly higher EE scores compared to healthy weight females (35.7 ± 23.7 vs. 11.9 ± 15.6, *p* = 0.002). No such observation was observed among overweight and obese males (n = 13 (18.8%), *p* > 0.05).

Given the absence of differences in outcomes between males and females described above, we sought to further confirm that sex did not influence TFEQ scores. To do this, a median split was used to classify participants as “high” or “low” on TFEQ factors. For example, those who scored less than the median score for RE were classified as low RE. Data were analyzed using a Fischer’s exact test. Membership in the high and low categories did not differ by sex for any of the TFEQ outcomes (Table 2). The only difference in outcomes was observed between members of the high and low UE groups who differed in their EE scores (10.4 ± 15.1 vs. 22.8 ± 18.8 (*p* < 0.001)). No other differences in TFEQ outcomes were present for the high and low EE groups or the high and low RE groups for any of the health outcomes after FDR.

## 4. Discussion

This study examined the relationships between eating behaviors as measured by the TFEQ-18 and physical and mental health outcomes. No differences were observed between males and females in terms of TFEQ mean subscale scores, but associations between TFEQ subscale scores and health outcomes did vary between the two groups. EE was associated with UE and BMI among males while EE was associated with UE, anxiety, and sleep quality among females. UE was only associated with EE, and RE was not associated with any health outcome for either sex. As with mean TFEQ subscale scores, no sex differences among health outcomes were observed. Differences in TFEQ scores were observed by BMI category only among females; women with overweight or obesity reported higher EE scores. Of the three subscales, EE was the only one to be associated with any of the health outcomes in this study.

The fact that EE was the only subscale associated with health outcomes could stem from the questionable internal consistency demonstrated by the UE subscale (Cronbach’s alpha = 0.610) and poor internal consistency demonstrated by the CR subscale (Cronbach’s alpha = 0.371) in our study population. While a number of statisticians have urged caution when using rule of thumb cut-off scales to assess reliability, e.g., [32,33]; other researchers who explored the psychometric properties of the TFEQ tool in their distinct populations have noted that the CR subscale is not reliable [34,35,36,37] and, in some cases, modified the questions within the CR subscale to improve reliability [34,35]. The evaluation of the psychometric properties of the TFEQ-18 in the Ghanaian population is outside the scope of this brief communication, but this is worthy of exploration in a more representative sample, and the questions regarding reliability, particularly with CR, should be used to temper conclusions based on the results presented.

Differences in TFEQ scores by sex are mixed based on the available literature. Multiple studies suggest females more frequently engage in EE, UE, and RE [26,27,28,29,38]. A previous study suggests EE scores were higher among female Ghanaian university students compared to males [17], but studies in other countries have reported no differences in any of the TFEQ subscale scores by gender [39,40]. Reasons for the discrepancy between the previous Ghanaian study and the current study are not immediately clear but might reflect differences due to COVID-19, as the present study collected data during this time period. However, Ghanaian students reported a lower incidence of increased stress due to the COVID-19 pandemic compared to students in many other countries, and the majority of respondents reported no change or reduced stress during the survey period based on our previous work [41]. Further work is needed to assess if differences in TFEQ scores truly exist among Ghanaian men and women.

While multiple studies report that BMI and EE, UE, and RE are positively correlated [18,42,43], others indicate that these behaviors are only weakly associated with BMI, if at all [38]. UE and RE were not associated with BMI for either sex in our study; however, EE and BMI were positively associated among males. Frequent engagement in emotional eating has been associated with higher adiposity and unhealthy dietary habits [18,42,44]. In our previous survey of college students from 7 countries, EE was associated with BMI for the entire student pool [45], which included Ghanaian students. In that study, males and females in the study population differed in EE and RE scores as well, with females reporting higher values for both. Further, results from the previous study indicated that the relationship between perceived stress and BMI was mediated by emotional eating for both males and females and suggests EE is a maladaptive stress coping response. Given that the Ghanaian sample in that study comprised approximately 9% of the respondents, it is quite possible that differences between Ghanaian students and the overall sample were lost. Further, some studies indicate that relationships between BMI and TFEQ subscales are dependent on adiposity, e.g., [38,46]; since our population’s mean BMI classifies them as lean, the utility of the TFEQ in predicting outcomes in this healthy group may be limited.

Relationships between EE, anxiety, and sleep quality were observed among females and agree with other reports. This prompts the question: why might EE be related to BMI, anxiety, and sleep quality? One possible connection stems from the fact that poor sleep quality can impair emotional regulation, making one more reactive to positive and negative emotions [47] and anxiety [48,49]. This increased emotional volatility could plausibly contribute to increased frequency of EE, and eating for emotional reasons while ignoring hunger and fullness cues, can contribute to excess energy intake and lead to obesity [50]. Poorer sleep quality has been shown to correlate with higher EE frequency, as measured by the Dutch Eating Behavior Questionnaire [51], and anxiety and sleep problems frequently occur together [52]. Thus, EE, anxiety, and sleep quality are plausibly connected.

Mindfulness training could be an appropriate intervention to address EE, anxiety, and sleep quality. For example, individuals who had higher mindfulness skills were less likely to engage in emotional eating as assessed by the TFEQ [53], and mindfulness interventions can improve sleep quality [54] as well as reduce anxiety [55]. For these reasons, testing whether mindfulness training can decrease emotional eating as well as anxiety while improving sleep outcomes is worthy of further study.

While the UE and RE scores for this study are comparable to our previous work with a large multi-country student population, EE scores among the Ghanaian students in this study are approximately half of the larger population surveyed [45]. Explanations for why this might be are beyond the scope of this study; however, the majority of Ghanaian students experienced no change or reduced levels of stress and anxiety during the data collection period [41], and as we suggested earlier, EE may provide means to cope with high levels of stress. Therefore, relatively low stress levels could have reduced the likelihood that EE would be used to manage stress. This supposition requires further testing.

### Strengths and Limitations

There are a number of strengths and limitations that should be noted when interpreting these results. This study used validated tools to assess outcomes; however, as the design was cross-sectional, causality cannot be determined. The study population was limited to one university in Ghana. For better generalizability, sampling from other universities should be undertaken. Data were collected during the COVID-19 pandemic; however, we previously reported that the majority of students experienced no change or reduced levels of stress and anxiety [41]. As mentioned above, the reliability of the CR subscale is well below commonly used cut-offs for acceptability, which suggest this subscale of the TFEQ-18 might not be appropriate to use in the Ghanaian population. Finally, while validated tools were used, not all tools were tailored specifically to the Ghanaian experience. This is an important consideration when interpreting results. For example, lower levels of dietary restraint have been observed among Ghanaian female college students compared to their American counterparts [56]; although, a different tool to assess restraint was used. These differences may be attributed, in part, to cultural differences that favor higher BMI among Ghanaians compared to Americans [56]. Thus, it is important to view these results through an appropriate cultural lens [38], and future work is needed to confirm or adapt the instruments used to ensure utility and validity in the Ghanaian population.

## 5. Conclusions

While absolute differences between sexes were not present, relationships between EE and health outcomes differed between males and females. EE was the only one of the three TFEQ-18 subscales to be associated with any of the health outcomes in this study: BMI for males and anxiety and sleep quality for females. While this study provides important information about the eating behaviors of Ghanaian university students and allows for comparison to students from other cultures, future work should ensure the validity of the tools used for the Ghanaian population.

## Figures and Tables

**Table 1:** Outcomes of interest by gender.

Outcome (Measure)	Males (n = 72)	Females (n = 57)	*p*-Value
BMI (kg/m^2^)	22.0 ± 4.9	24.3 ± 7.1	0.054
Emotional Eating (TFEQ)	15.3 ± 14.6	17.8 ± 21.7	0.426
Uncontrolled Eating (TFEQ)	27.4 ± 11.8	23.7 ± 17.4	0.173
Restrained Eating (TFEQ)	27.8 ± 13.8	35.2 ± 17.1	0.007
Dietary Patterns (STC)	6.2 ± 2.5	6.9 ± 2.7	0.153
Perceived Stress (PSS-10)	19.1 ± 5.7	20.6 ± 6.4	0.155
Financial Stress (USFSA)	16.3 ± 4.7	14.6 ± 5.0	0.048
Resilience (BRS)	3.3 ± 0.7	3.1 ± 0.8	0.061
Generalized Anxiety (GAD-7)	6.0 ± 4.8	7.7 ± 6.4	0.102
Sleep Quality (PSQI)	5.7 ± 2.7	6.1 ± 3.6	0.448
Sleep Duration—Weekday (h)	6.7 ± 1.7	6.5 ± 1.7	0.381
Sleep Duration—Weekend (h)	7.3 ± 2.1	6.7 ± 2.1	0.104

After false discovery rate correction, no differences between males and females were observed. The *p*-value represents the unadjusted *p*-value to aid in interpretation. Missing data for BMI: males n = 11, females n = 10. Abbreviations: BMI: body mass index, TFEQ: Three Factor Eating Questionnaire, STC: Starting the Conversation, PSS-10: Perceived Stress Scale-10, USFSA: University Student Financial Stress Assessment, BRS: Brief Resilience Scale, GAD-7: Generalized Anxiety Disorder Screener-7, PSQI: Pittsburgh Sleep Quality Index. Sleep duration was self-reported.

**Table 2:** Median scores by TFEQ subscale.

Outcome (Measure)	Median ± SIQR	*p*-Value
Emotional Eating (TFEQ)	8.3 ± 12.5	>0.999
Uncontrolled Eating (TFEQ)	25.0 ± 11.1	0.376
Restrained Eating (TFEQ)	29.2 ± 11.5	0.860

Data presented as median ± semi-interquartile range (SIQR). *p*-value reflects Fischer’s Exact Test for differences between males and females. Participants below median (n = 63). Participants above median (n = 66).

## Data Availability

Data can be obtained by contacting the corresponding author.

## References

[1] Martins B.G., da Silva W.R., Maroco J., Campos J.A.D.B. (2021). Psychometric characteristics of the Three-Factor Eating Questionnaire-18 and eating behavior in undergraduate students. Eat. Weight Disord. Stud. Anorex. Bulim. Obes..

[2] Chen G.-Q., Peng C.-L., Lian Y., Wang B.-W., Chen P.-Y., Wang G.-P. (2021). Association between Dietary Inflammatory Index and mental health: A systematic review and dose–response meta-analysis. Front. Nutr..

[3] Firth J., Marx W., Dash S., Carney R., Teasdale S.B., Solmi M., Stubbs B., Schuch F.B., Carvalho A.F., Jacka F. (2019). The effects of dietary improvement on symptoms of depression and anxiety: A meta-analysis of randomized controlled trials. Psychosom. Med..

[4] de Lauzon B., Romon M., Deschamps V., Lafay L., Borys J.-M., Karlsson J., Ducimetière P., Charles M.A., Fleurbaix Laventie Ville Sante Study Group (2004). The Three-Factor Eating Questionnaire-R18 is able to distinguish among different eating patterns in a general population. J. Nutr..

[5] Stunkard A.J., Messick S. (1985). The Three-Factor Eating Questionnaire to measure dietary restraint, disinhibition and hunger. J. Psychosom. Res..

[6] Karlsson J., Persson L.O., Sjostrom L., Sullivan M. (2000). Psychometric properties and factor structure of the Three-Factor Eating Questionnaire (TFEQ) in obese men and women. Results from the Swedish Obese Subjects (SOS) study. Int. J. Obes. Relat. Metab. Disord. J. Int. Assoc. Study Obes..

[7] Konttinen H. (2020). Emotional eating and obesity in adults: The role of depression, sleep and genes. Proc. Nutr. Soc..

[8] Liu H., Yang Q., Luo J., Ouyang Y., Sun M., Xi Y., Yong C., Xiang C., Lin Q. (2020). Association between emotional eating, depressive symptoms and laryngopharyngeal reflux symptoms in college students: A cross-sectional study in Hunan. Nutrients.

[9] Chearskul S., Pummoung S., Vongsaiyat S., Janyachailert P., Phattharayuttawat S. (2010). Thai version of Three-Factor Eating Questionnaire. Appetite.

[10] Kristanto T., Chen W.S., Thoo Y.Y. (2016). Academic burnout and eating disorder among students in Monash University Malaysia. Eat. Behav..

[11] Löffler A., Luck T., Then F.S., Sikorski C., Kovacs P., Böttcher Y., Breitfeld J., Tönjes A., Horstmann A., Löffler M. (2015). Eating behaviour in the general population: An analysis of the factor structure of the German version of the Three-Factor-Eating-Questionnaire (TFEQ) and its association with the body mass index. PLoS ONE.

[12] Hainer V., Kunesova M., Bellisle F., Parizkova J., Braunerova R., Wagenknecht M., Lajka J., Hill M., Stunkard A. (2006). The Eating Inventory, body adiposity and prevalence of diseases in a quota sample of Czech adults. Int. J. Obes..

[13] Lopez-Cepero A., Frisard C.F., Lemon S.C., Rosal M.C. (2018). Association of dysfunctional eating patterns and metabolic risk factors for cardiovascular disease among Latinos. J. Acad. Nutr. Diet..

[14] Shiozawa K., Mototani Y., Suita K., Ito A., Matsuo I., Hayakawa Y., Kiyomoto K., Tsunoda M., Nariyama M., Umeki D. (2020). Gender differences in eating behavior and masticatory performance: An analysis of the Three-Factor-Eating Questionnaire and its association with body mass index in healthy subjects. J. Oral Biosci..

[15] Lesdéma A., Fromentin G., Daudin J.-J., Arlotti A., Vinoy S., Tome D., Marsset-Baglieri A. (2012). Characterization of the Three-Factor Eating Questionnaire scores of a young French cohort. Appetite.

[16] Keskitalo K., Tuorila H., Spector T.D., Cherkas L.F., Knaapila A., Kaprio J., Silventoinen K., Perola M. (2008). The Three-Factor Eating Questionnaire, body mass index, and responses to sweet and salty fatty foods: A twin study of genetic and environmental associations. Am. J. Clin. Nutr..

[17] Intiful F.D., Oddam E.G., Kretchy I., Quampah J. (2019). Exploring the relationship between the big five personality characteristics and dietary habits among students in a Ghanaian university. BMC Psychol..

[18] de Lauzon-Guillain B., Basdevant A., Romon M., Karlsson J., Borys J.-M., Charles M.A., The FLVS Study Group (2006). Is restrained eating a risk factor for weight gain in a general population?. Am. J. Clin. Nutr..

[19] Paxton A.E., Strycker L.A., Toobert D.J., Ammerman A.S., Glasgow R.E. (2011). Starting the Conversation: Performance of a brief dietary assessment and intervention tool for health professionals. Am. J. Prev. Med..

[20] Cohen S., Kamarck T., Mermelstein R. (1983). A global measure of perceived stress. J. Health Soc. Behav..

[21] Lim H., Heckman S.J., Letkiewicz J.C., Montalto C.P. (2014). Financial stress, self-efficacy, and financial help-seeking behavior of college students. J. Financ. Couns. Plan..

[22] Smith B.W., Dalen J., Wiggins K., Tooley E., Christopher P., Bernard J. (2008). The Brief Resilience Scale: Assessing the ability to bounce back. Int. J. Behav. Med..

[23] Löwe B., Decker O., Müller S., Brähler E., Schellberg D., Herzog W., Herzberg P.Y. (2008). Validation and standardization of the Generalized Anxiety Disorder Screener (GAD-7) in the general population. Med. Care.

[24] Buysse D.J., Reynolds III C.F., Monk T.H., Berman S.R., Kupfer D.J. (1989). The Pittsburgh Sleep Quality Index: A new instrument for psychiatric practice and research. Psychiatry Res..

[25] Yeboah K., Dodam K.K., Agyekum J.A., Oblitey J.N. (2022). Association between poor quality of sleep and metabolic syndrome in Ghanaian university students: A cross-sectional study. Sleep Disord..

[26] Davis E., Greenberger E., Charles S., Chen C., Zhao L., Dong Q. (2012). Emotion experience and regulation in China and the United States: How do culture and gender shape emotion responding?. Int. J. Psychol..

[27] De Lauzon-Guillain B., Romon M., Musher-Eizenman D., Heude B., Basdevant A., Charles M.A., Fleurbaix-Laventie Ville Santé Study Group (2009). Cognitive restraint, uncontrolled eating and emotional eating: Correlations between parent and adolescent. Matern. Child. Nutr..

[28] Kemp E., Bui M., Grier S. (2011). Eating their feelings: Examining emotional eating in at-risk groups in the United States. J. Consum. Policy.

[29] Thompson S.H. (2015). Gender and racial differences in emotional eating, food addiction symptoms, and body weight satisfaction among undergraduates. J. Diabetes Obes..

[30] Cronbach L.J. (1951). Coefficient alpha and the internal structure of tests. Psychometrika.

[31] Benjamini Y., Hochberg Y. (1995). Controlling the false discovery rate: A practical and powerful approach to multiple testing. J. R. Stat. Soc. Ser. B Methodol..

[32] Schmitt N. (1996). Uses and abuses of coefficient alpha. Psychol. Assess..

[33] Vaske J.J., Beaman J., Sponarski C.C. (2017). Rethinking internal consistency in Cronbach’s alpha. Leis. Sci..

[34] Wrzecionkowska D., Rivera Aragón S., Wrzecionkowska D., Rivera Aragón S. (2021). Three-Factor Eating Questionnaire-R18 (TFEQ-R18) Spanish Version: Factor Structure Analysis among Normal Weight and Overweight Adults. Acta Investig. Psicológica.

[35] Halali F., Lapveteläinen A., Karhunen L., Kantanen T. (2020). Eating behavior tendencies among Finnish adults in relation to previous weight loss attempts. Appetite.

[36] Chong M.F.-F., Ayob M.N.M., Chong K.J., Tai E.-S., Khoo C.M., Leow M.K.-S., Lee Y.S., Tham K.W., Venkataraman K., Meaney M.J. (2016). Psychometric analysis of an eating behaviour questionnaire for an overweight and obese Chinese population in Singapore. Appetite.

[37] Alhebshi S., Hilary S., Safi S., Ali H.I., Ismail L.C., Al-Dhaheri A., Stojanovska L. (2022). Validity and Reliability of Arabic Version of the Three-Factor Eating Questionnaire-18. https://papers.ssrn.com/sol3/papers.cfm?abstract_id=4188501.

[38] Cappelleri J.C., Bushmakin A.G., Gerber R.A., Leidy N.K., Sexton C.C., Lowe M.R., Karlsson J. (2009). Psychometric analysis of the Three-Factor Eating Questionnaire-R21: Results from a large diverse sample of obese and non-obese participants. Int. J. Obes..

[39] Boerner L.M., Spillane N.S., Anderson K.G., Smith G.T. (2004). Similarities and differences between women and men on eating disorder risk factors and symptom measures. Eat. Behav..

[40] Gallant A.R., Tremblay A., Pérusse L., Bouchard C., Després J.-P., Drapeau V. (2010). The Three-Factor Eating Questionnaire and BMI in adolescents: Results from the Québec Family Study. Br. J. Nutr..

[41] Adjepong M., Amoah-Agyei F., Du C., Wang W., Fenton J.I., Tucker R.M. (2022). Limited negative effects of the COVID-19 pandemic on mental health measures of Ghanaian university students. J. Affect. Disord. Rep..

[42] Konttinen H., Van Strien T., Männistö S., Jousilahti P., Haukkala A. (2019). Depression, emotional eating and long-term weight changes: A population-based prospective study. Int. J. Behav. Nutr. Phys. Act..

[43] Neumark-Sztainer D., Wall M., Haines J., Story M., Eisenberg M.E. (2007). Why does dieting predict weight gain in adolescents? Findings from project EAT-II: A 5-year longitudinal study. J. Am. Diet. Assoc..

[44] Beydoun M.A., Gary T.L., Caballero B.H., Lawrence R.S., Cheskin L.J., Wang Y. (2008). Ethnic differences in dairy and related nutrient consumption among US adults and their association with obesity, central obesity, and the metabolic syndrome. Am. J. Clin. Nutr..

[45] Du C., Adjepong M., Zan M.C.H., Cho M.J., Fenton J.I., Hsiao P.Y., Keaver L., Lee H., Ludy M.-J., Shen W. (2022). Gender differences in the relationships between perceived stress, eating behaviors, sleep, dietary risk, and body mass index. Nutrients.

[46] Harden C.J., Corfe B.M., Richardson J.C., Dettmar P.W., Paxman J.R. (2009). Body mass index and age affect Three-Factor Eating Questionnaire scores in male subjects. Nutr. Res..

[47] Gruber R., Cassoff J. (2014). The interplay between sleep and emotion regulation: Conceptual framework empirical evidence and future directions. Curr. Psychiatry Rep..

[48] Ramsawh H.J., Stein M.B., Belik S.-L., Jacobi F., Sareen J. (2009). Relationship of anxiety disorders, sleep quality, and functional impairment in a community sample. J. Psychiatr. Res..

[49] Spira A.P., Stone K., Beaudreau S.A., Ancoli-Israel S., Yaffe K. (2009). Anxiety symptoms and objectively measured sleep quality in older women. Am. J. Geriatr. Psychiatry.

[50] Lowe M.R., Fisher E.B. (1983). Emotional reactivity, emotional eating, and obesity: A naturalistic study. J. Behav. Med..

[51] Dweck J.S., Jenkins S.M., Nolan L.J. (2014). The role of emotional eating and stress in the influence of short sleep on food consumption. Appetite.

[52] Alvaro P.K., Roberts R.M., Harris J.K. (2013). A systematic review assessing bidirectionality between sleep disturbances, anxiety, and depression. Sleep.

[53] Pidgeon A., Lacota K., Champion J. (2013). The moderating effects of mindfulness on psychological distress and emotional eating behaviour. Aust. Psychol..

[54] Rusch H.L., Rosario M., Levison L.M., Olivera A., Livingston W.S., Wu T., Gill J.M. (2019). The effect of mindfulness meditation on sleep quality: A systematic review and meta-analysis of randomized controlled trials. Ann. N. Y. Acad. Sci..

[55] Hofmann S.G., Gómez A.F. (2017). Mindfulness-based interventions for anxiety and depression. Psychiatr. Clin. N. Am..

[56] Cogan J.C., Bhalla S.K., Sefa-Dedeh A., Rothblum E.D. (1996). A comparison study of United States and African students on perceptions of obesity and thinness. J. Cross-Cult. Psychol..

